# Supplementary material for: NN-assisted image analysis for quantifying intracellular Trypanosoma cruzi infection
Source: Front Cell Infect Microbiol. 2026 Aug 6;16:1885877. doi: 10.3389/fcimb.2026.1885877 (PMC13489841; doi:10.3389/fcimb.2026.1885877)
Supplement: Supplementary file 1 [file Supplementaryfile1.pdf]

Supporting information

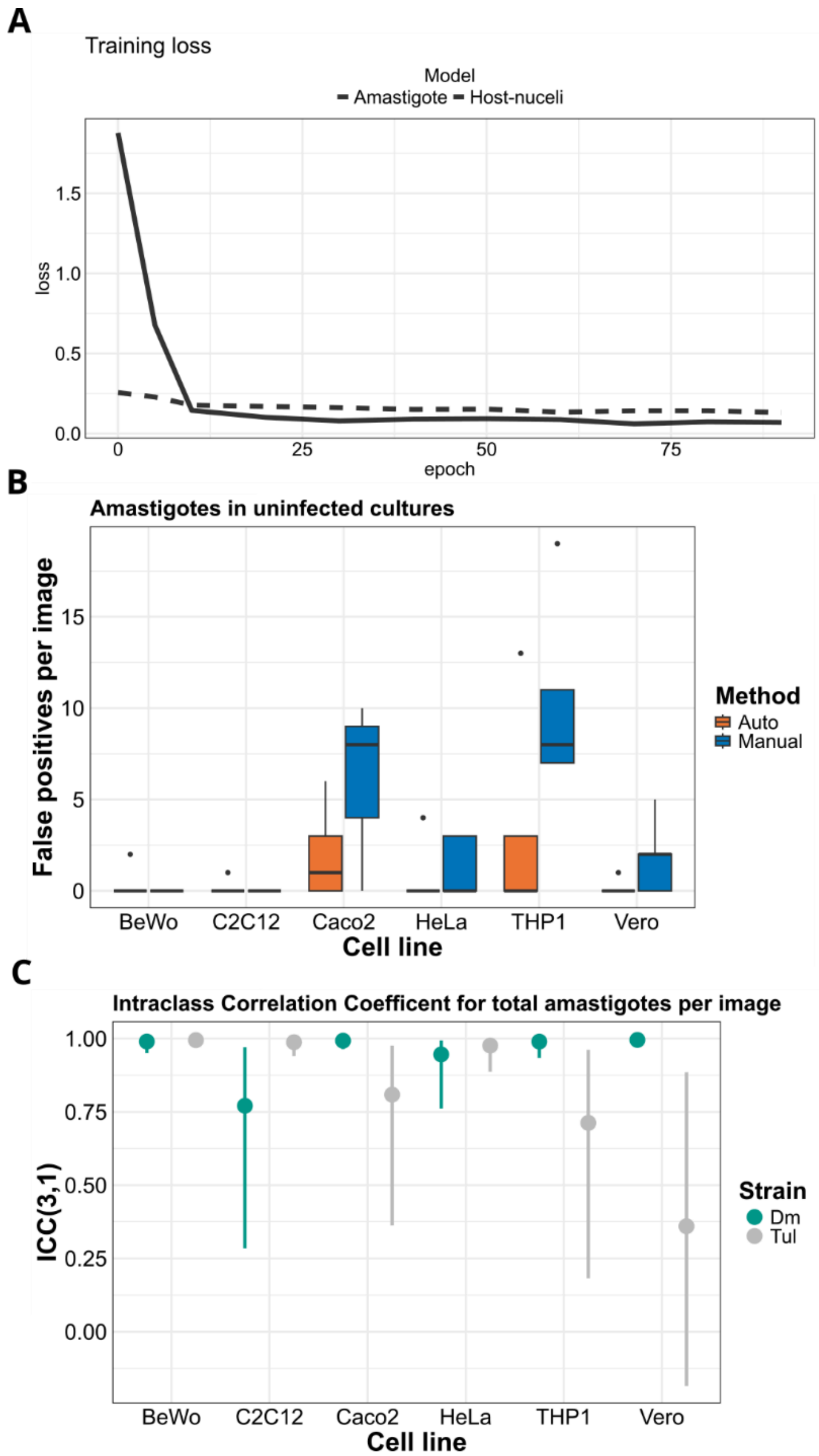

Figure S1. A: Training loss during the fine-tuning of the mammalian cell (dashed line) and amastigote (full line) models on their complete training datasets, for 100 epochs. B: Number of amastigotes detected by a human operator or the automated algorithm in images of uninfected cultures. C: Two-way mixed, single measurement Intraclass Correlation Coefficient (ICC(3,1)) for two human operators across all experimental conditions in the validation dataset.

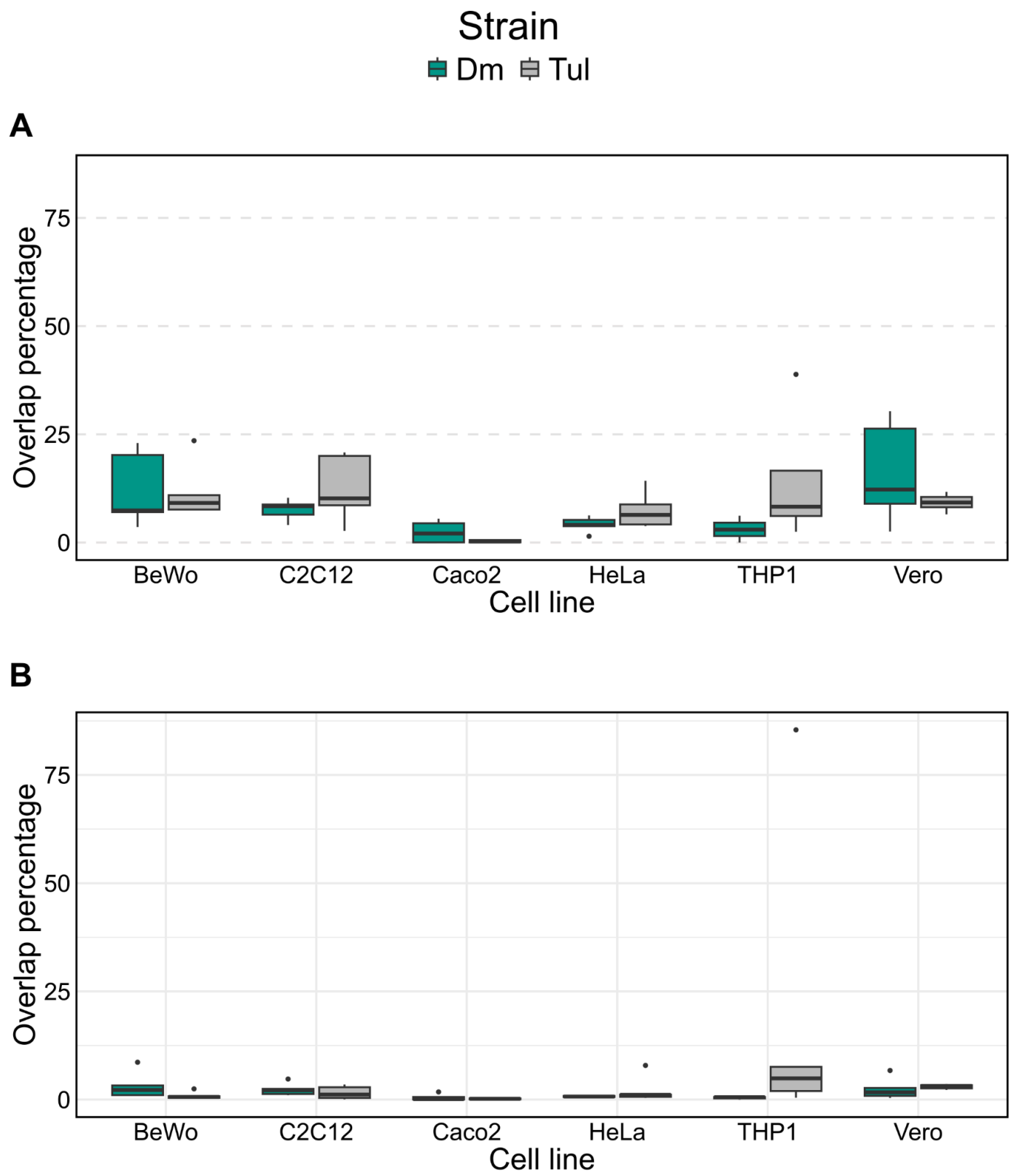

Figure S2. Box plots for the percentage of the total surface area of the host-nuclei masks that overlapped with the specific signal form the anti-*T. cruzi* serum, for each image in the Cell line-Strain group. A: masks were generated with the Cellpose-SAM model. B: masks were generated with the refined model. Dm: infection with Dm28c trypomastigotes. Tul: infection with Tulahuen trypomastigotes.

Cell line	Parasite strain	Microscopy	Magnification	Images
BeWo	Dm	Confocal	60	2
C2C12	Dm	Widefield	20	8
C2C12	Dm	Confocal	60	5
Caco2	Dm	Confocal	60	3
THP1	Dm	Confocal	60	1
Vero	Dm	Confocal	40	2
Vero	Dm	Confocal	60	2
BeWo	K98	Widefield	20	7
Vero	K98	Widefield	20	7
BeWo	Tul	Confocal	40	1
BeWo	Tul	Confocal	60	1
BeWo	Tul	Widefield	40	13
C2C12	Tul	Widefield	20	3
Caco2	Tul	Confocal	40	2
HeLa	Tul	Confocal	60	2
THP1	Tul	Confocal	60	6
Vero	Tul	Confocal	60	1
Vero	Tul	Widefield	40	6
Vero	Tul	Confocal	40	1
Vero	Y	Widefield	20	3

Table S1. Summary of the images that were part of the dataset used to fine-tune the model to detect host-cell nuclei.

Cell line	Parasire strain	Microscopy	Magnification	Images
BeWo	Dm	Confocal	60	3
BeWo	Tul	Confocal	60	1
BeWo	K98	Widefield	40	7
BeWo	Tul	Confocal	40	12
C2C12	Dm	Widefield	20	6
C2C12	Tul	Widefield	20	4
C2C12	Dm	Confocal	60	5
Caco2	Dm	Confocal	60	6
Caco2	Tul	Confocal	40	1
HeLa	Tul	Confocal	60	1
HeLa	Dm	Confocal	60	5
HeLa	Tul	Confocal	40	2
THP1	Dm	Confocal	60	3
Vero	Tul	Confocal	60	4
Vero	Uninfected	Confocal	60	10
Vero	Y	Widefield	40	3
Vero	Dm	Confocal	40	2
Vero	Dm	Confocal	60	1
Vero	Tul	Confocal	40	3
Vero	K98	Widefield	40	8

Table S2. Summary of the images that were part of the dataset used to fine-tune the model to detect *T. cruzi* amastigotes.

Cell line	Parasite strain	Microscopy	Magnification	Images
BeWo	Dm	Confocal	60	5
BeWo	Tul	Confocal	60	5
C2C12	Dm	Confocal	60	5
C2C12	Tul	Confocal	60	5
Caco2	Dm	Confocal	60	5
Caco2	Tul	Confocal	60	5
HeLa	Dm	Confocal	60	5
Vero	Dm	Confocal	60	5
Vero	Tul	Confocal	60	5
HeLa	Tul	Confocal	60	5
THP-1	Dm	Confocal	60	5
THP-1	Tul	Confocal	60	4

Table S3. Summary of the images used in the validation dataset.
